# The role of individual service and team-based service price in the online environment: A view from the price difference

**DOI:** 10.3389/fpubh.2022.935613

**Published:** 2022-10-17

**Authors:** Wei Lu, Wei Wei, Chao Li, Qing Luo, Lichun Fan

**Affiliations:** ^1^School of Management, Hainan Medical University, Haikou, China; ^2^Hainan Women and Children's Medical Center, Haikou, China

**Keywords:** service price, price difference, team-based service, virtual team, online medical platform

## Abstract

Different from the traditional medical market, the online medical market allows physicians considerable discretion in setting prices of their services, which is beginning to be paid close attention to. Physicians face a challenge with the introduction of various service styles. Guided by transaction utility theory and price fairness, this study aims to investigate the influence of pricing strategy on service demands from the price difference perspective by focusing on two typical service models: individual service and team-based service. Moreover, team characteristics (response speed and team size) are also considered. The data collection was done in March 2018 and repeated in May 2018, and physicians who provide both individual service and team-based services are included in our study. Finally, a dataset consisting of 1,100 teams with 1,100 physician leaders from 14 departments such as obstetrics and gynecology department were collected from an online medical platform in China. Empirical results support most of our hypotheses. A negative influence of team-based price was observed. As a substitute service, a higher individual service price will make patients turn to team-based service. Moreover, individual service prices negatively moderated the relationship between team-based service prices and demands. By calculating the price difference between the individual service price and the team-based service price, we found a negative role of the price difference affecting patient purchase decisions. Although we did not find a significant effect of team size, a quick response can attract more patients. Price fairness provides a proper framework for understanding pricing strategy in individual and team-based service in an online environment. Understanding the effects of prices from a price difference perspective has both theoretical and practical contributions. Specifically, this study contributes to knowledge on price fairness, online medical platforms, and virtual teams, and provides management suggestions.

## Introduction

“*Pricing Flexibility—you are free to set your own pricing for your services*.”

Pricing strategy in the traditional environment cannot reflect the value of the physicians' medical services, leading in turn to patient-physician conflict to a certain extent. In recent years, online medical services have gained rapid and wide development. Up to a point, the reasons behind this are obvious from the physician's perspective: market-regulated prices that the service price determined autonomously by the physicians and formed through market competition can better reflect their service value, resulting in improved productivity and retention (1). Based on the policy promotion of consumption upgrading and the reality of a lack of high-quality medical services in China, online paid medical services are a feasible source of income for physicians at the present stage and promise a trend of continuous growth.

In marketing, the price has been crucial to consumer buying decisions (2) and switching service providers (3), whether online or offline. The easy availability of information and lower search costs on the Internet enables consumers to compare different products and price information more efficiently and make informed decisions (4). The dual role of price in influencing consumer behaviors has been recognized, namely a negative role for giving up or a positive role to acquire (5, 6). In a highly uncertain context, price is often treated as a cue for quality or benefit (7).

These complex effects have also entered healthcare. Recently, with the development of online medical services, researchers have begun to look at the role of price in patients' decisions. When facing a serious disease, patients tend to choose physicians with a high service fee, while the negative influence has been universally obtained based on transaction cost theory for patients with mild diseases (8). In addition, the price has a non-linear relationship with patient satisfaction (9). However, current studies mainly focus on the physician's individual services. This paper focuses on patient decisions on team-based service.

Team-based services are highly regarded for their advantages compared to individual services and have become an integral part of healthcare organizations (10). A team, which is embedded in an organization or context, can be defined as a social system of three or more people who collaborate on a common task (11). Good teamwork brings good outcomes, such as comprehensive and rapid treatment in healthcare (12). Different from the offline medical team in hospitals, the online medical team has broader cooperation *via* the Internet, where all kinds of people related to the patient's medical process can join in and give guidance to the patient (13).

The theory of transaction utility explains consumer satisfaction regarding the price paid in comparison to an established reference or anchor price (14). Consumers encode and interpret actual prices in ways that are meaningful to them (15). They have adaptation level prices or latitude of acceptable prices for a given product category (16). They often judge the actual price of a product in comparison with these internal standards (6). This suggests that it is the perceived product price rather than the actual price that affects consumers' product evaluation and choices (15). In the online medical environment, individual service and team-based service have the same service form (i.e., text consultation), while their service providers overlap partly. Therefore, patients' decisions might be influenced by both the price of the individual service and the price of the team-based service, and their price difference. This prompts the research question: how should physicians set prices for individual service and team-based service?

With the development of the economy, consumers are increasingly seeking a variety of products or services, therefore, the need for a better understanding of the effects of prices from a price difference perspective is supported by both managerial and theoretical reasons. From a managerial perspective, there are questions about how the price influences patient decisions on the Internet and how online patients perceive the prices of various services from the same provider. From a theoretical perspective, it's necessary to go beyond the extant studies that have focused on the role of price in healthcare to gain insights into how the price difference of various services affects decision-making.

## Theoretical background

### Transaction utility theory

Behavioral economics suggests that individuals attempt to maximize utility in transactions. To examine all possibilities to maximize utility is unrealistic, so individuals try to find an acceptable outcome that may not be the optimal solution (17), namely “satisficing alternative.”

Based on transaction utility theory (14), the perceived value of a transaction is influenced by more than just the price, it is also influenced by other factors such as attitudes, seller image, experiences, and location. These factors jointly create a reference price in the consumer's mind and then would be used as a point of comparison to the price. Transaction utility focuses on an individual's assessment of a certain price and value (18). It suggests that a price below an established point of reference produces a positive transaction value which is more likely to lead to an actual purchase, whereas a price above a reference point produces a negative transaction value which is much less likely to lead to a purchase. Price above a reference point might be perceived as a lack of fairness.

In terms of medical services, fairness perceptions may be influenced by factors such as the accessibility of medical resources, medical skills, bedside manner, or comparisons to a reference price such as the price of other physicians in the same department and other service types of the focus physician. In addition, disease types, and patient attitudes could play a role in fairness perceptions.

### Price fairness

The concept of fairness is a significant factor in consumer evaluations (19). It is defined as consumer perceptions and related emotions (i.e., fair, acceptable, and reasonable) on the difference between two prices (20).

There are two main research streams for existing studies on price fairness. The first one aims to identify factors that influence the perceptions of price fairness (21). The other one focus on the consequence of price fairness (19). Price fairness influences consumers' trust and satisfaction with the service provider (22). If consumers perceive that they are being overcharged, they will not trust and will be more likely to switch to another service provider (23). Deviation from this reference or anchor price might be perceived as unfair and decreases purchase (24). Price fairness influences not only the intention to buy but also some forms of negative behaviors that directly harm the seller, e.g., negative word of mouth, complaints, and leaving the seller (19).

A reference point is any kind of stimulus with known characteristics that embody standards to which people compare categorically similar stimuli with unknown characteristics in an attempt to acquire new information (25). The higher the resemblance of reference points, the higher the inclination to make comparisons (26). These reference points may be represented by exact product prices (context-based). The choice of any reference point is based on its availability and similar others are the most salient reference points in the transaction environment (27).

## Hypotheses development

**Figure 1:**
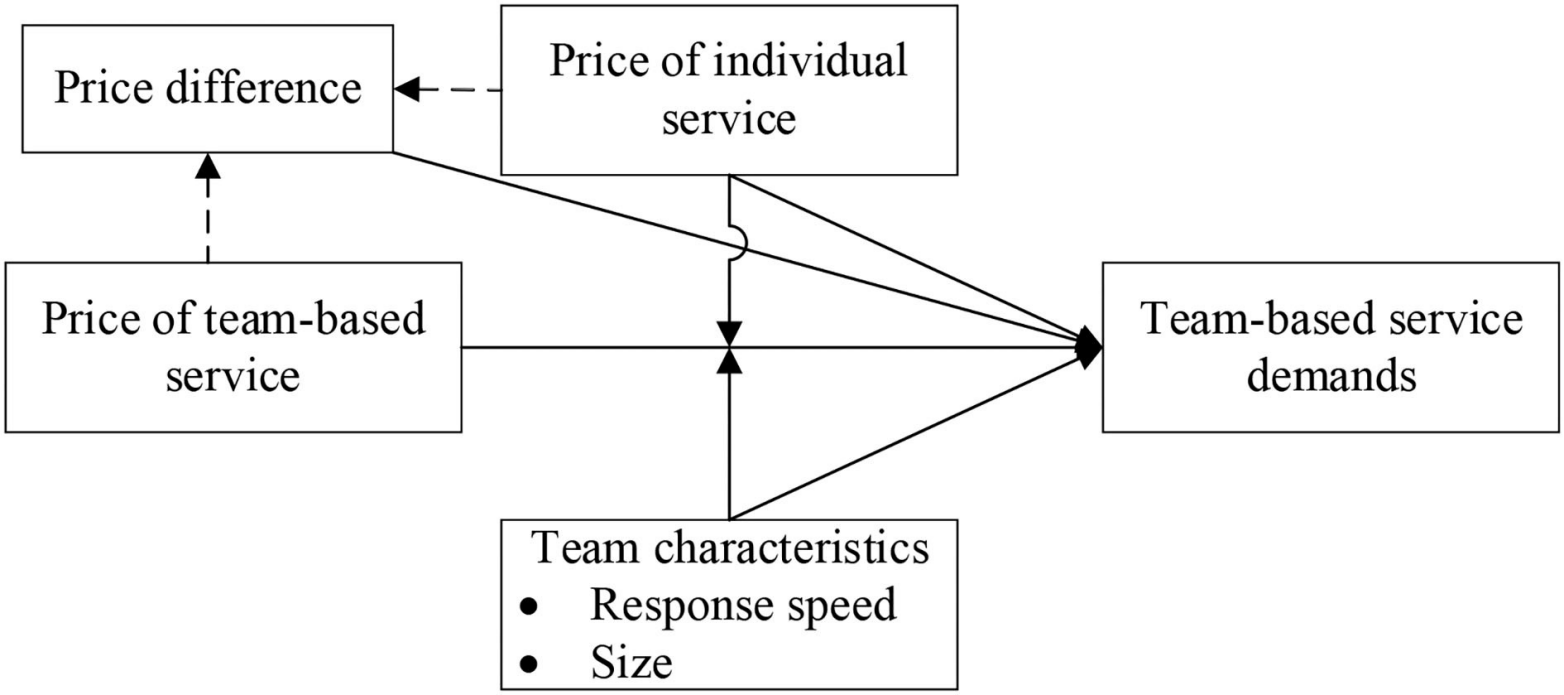
Conceptual model.

In an online medical environment, a physician can provide individual service and also can cooperate with other physicians to provide team-based service. Therefore, individual service and team-based service have the same service form (i.e., text consultation), but their service providers overlap partly. Different from existing studies, we focus on team-based service demands and aim to examine the influences of the individual service price, team-based service price, and their price difference on patient purchase decisions toward team-based service. The conceptual model is shown in Figure 1.

### Team-based service price, individual service price, and team-based service demands

In marketing, price plays a dual role, an indicator of monetary sacrifice or a cue for quality (28). When price plays the sacrifice role, it would negatively influence consumer purchase, whereas, when price mainly plays the quality cue, it reflects the positive aspect of consumer decision. Therefore, to investigate the role of price, relative weights of the two roles of price need to be identified.

From the quality cue view, price is the monetary value placed on products or services offered by providers to their consumers. Prior studies have shown that a high price leads to a high-quality perception of products or services (29). The price-quality perception is a subjective expectation of a product or service. However, service quality, in general, involves higher variability and uncertainty than product quality and is therefore difficult to anticipate before purchase. Even after receiving the service, the consumer often still cannot evaluate the quality. Thus, price is not a pivotal cue to infer service quality; consequently, people may not employ the price-quality inference when evaluating service quality (30). More so, when relating to health and medical service, which makes it a special case.

In healthcare, patients often pursue high-quality medical resources at all costs as medical service has a direct relation with health and life for patients. A common phenomenon is that top-level hospitals and physicians are overwhelmed. With the development of online medical platforms, more information is provided publicly and can be used to judge quality. By exploring the role of price in the online medical environment, researchers have proven that when facing a serious disease, patients tend to choose physicians with a high service fee, while the negative influence has been universally obtained based on transaction cost theory for patients with mild diseases (8). However, current studies mainly focus on the physician's individual services.

Due to the lack of face-to-face contact, online medical services have shown the greatest promise in dealing with the most common health problems. It does not apply to patients who must undergo a medical examination, or to those with complicated diseases (31). Therefore, in this paper, we focus on these mild diseases and propose the following hypothesis based on existing studies:

**H1a:** *Team-based service price negatively impacts team-based service demands in the online environment*.

As individual service and team-based service have the same service form (i.e., text consultation), they are often substitutable. Based on classical price theory (32), when individual service price is high, patients are more likely to choose team-based service, leading to the following hypothesis.

**H1b:** *Individual service price positively impacts team-based service demands in the online environment*.

### The price difference and team-based service demands

Consumer decisions are often influenced by their expected price which is defined as the price which a consumer thinks he/she will have to pay for obtaining a defined product or service (29). It usually relates to prices consumers previously encountered (33), namely reference points. A reference point is any kind of stimulus with known characteristics that embody standards to which people compare categorically similar stimuli with unknown characteristics in an attempt to acquire new information (25). The higher the resemblance of reference points, the higher the inclination to make comparisons (26).

These reference points may be represented by exact product prices which are context-based. In our context, when a physician chooses to provide both individual service and team-based service, the individual service price becomes an important reference price for patients who are making decisions on team-based service. The service provider of the individual service is also a member of the team-based service and jointly provides service with other members to patients. As the choice of any reference point is based on its availability and similar others are the most salient reference points in the transaction environment (27), then individual service price would become an available reference point price.

As both individual service and team-based service are provided by the focus physician, patients' price perception would be influenced by them both. On the one hand, when the individual price is high, it improves patients' sensitivity to price, therefore the negative influence of team-based service improves. On the other hand, consider a situation where a patient is faced with two team-based services at the same price, but their individual service price is different. The patient might believe that behind the high price difference is the greatly reduced quality of service. Therefore, we propose a negative moderating effect on the individual service price.

**H2:** *Individual service price negatively moderates the relationships between team-based service price and team-based service demands*.

The reasonable difference between two prices influences consumer price fairness perceptions (20). Individuals evaluate the ratio between their own investment and its respective return, parallel to the ratio of the investment and return of another party enlisted in the exchange (34). Any significant difference between both ratios in favor of one or the other party leads to perceptions of unfairness (19). The big price difference between the individual service price and the team-based service price would enhance patients' unfairness perception and consequently lower their purchase intents.

**H3:** *Price difference negatively impacts patient demands for a team-based service*.

### Team characteristics and demands

Personalization reduces similarity across competing products or brands, thus, making a direct price comparison more difficult for consumers. Besides price, some other characteristics could influence patient decisions. Prior studies have proved that virtual team composition (e.g., team characteristics) significantly affects team performance (35–37).

Except for the price, response speed and team size also impact patient decisions directly. For patients, time is an important factor when they make decisions. Studies that focused on individual service have examined the relationship between response speed and patient satisfaction (38). For the team-based service studies, they found that team diversity (reputation diversity and experience diversity) of physicians in teams exert positive influences on team performance (39). Although team size (i.e., the number of team members) has consistently been regarded as a major antecedent of social loafing or productivity loss in technology-supported teams (40, 41), patients may prefer big-sized teams as there are more members to provide service. In addition, a big team size helps improve diversity. This study focuses on the moderating effects of team characteristics and proposes the following hypotheses.

**H4a:** *Response speed positively moderates the relationship between team-based service price and team-based service demands*.
**H4b:** *Team size positively moderates the relationship between team-based service price and team-based service demands*.

## Methods

### Research context

We collected data from Haodf, which is one of the most professional and largest online medical platforms in China. Although there are a few other popular online medical platforms such as Guahao and Chunyuyisheng, they are yet to provide team-based medical services. Haodf introduced the team-based medical service (called expert team) as a new service type in 2016. A team is formed spontaneously by a physician leader and several physician members who may come from different departments and hospitals. So far there are more than 3,300 teams with 10,000 physicians. Physicians and teams are free to set their service prices under the management of the platform. Given the above, this website was suitable for us to research pricing strategies in healthcare.

### Sample and data collection

Based on the department's classification rule of Haodf, we collected information on physicians and teams from 13 departments mainly from gynecology and obstetrics, endocrinology, E.N.T., and psychiatry departments using Python. The data were collected in March 2021 and repeated in May 2021. Finally, a dataset consisting of 1,100 teams with 1,100 physician leaders was included. In our study, individual service refers to the service of the team leader.

### Measurement

Table 1 provides the description and measurement of all variables in this study. The department variable in the empirical model is the team-based service demands (*#Demands*) which are measured by the increment of team-based service orders during the two collection times. Service orders reflect team performance and are important to team survival.

**Table 1:** Description of variables.

**Variables**		**Description**	**Measurement**
**Dependent variable**			
Team-based service demands	*#Demands*	The increment of orders of team-based service between March and May in 2018.	Its logarithm value.
**Independent and moderating variables**			
Team-based service price	*TSP*	The price of team-based service.	Its logarithm value.
Individual service price	*ISP*	The price of individual service, namely service of team leader.	Its logarithm value.
Price difference	*AbsPD*	The price difference between individual service price and team-based service price.	Its absolute value
Team-based response speed	*TRS*	The response speed of team-based service. The response speed is measured by the percentage that the patient can get a reply in 24 h.	Original data
Team members	*#Members*	The number of team members including the team leader.	Original data
**Control variables**			
**For team-**			
Team introduction	*Tint*	Introduction about the field of expertise on the team homepage.	The logarithm value of introduction words.
**For leader-**			
Hospital level	*Level*	The hospital level is evaluated by the government and presents the hospital's ability, equipment, and technology. It includes AAA, AA, and A level.	A dummy variable is used to measure whether the leader works in an AAA-level hospital.
Hospital introduction	*Hint*	Introduction about the history and experience of the hospital.	The logarithm value of introduction words.
City located	*City*	The level of economic development determines the residents' consumption ability.	A dummy variable is used to measure whether a leader works in a top-tier city.
Medical title	*Title*	The medical title is evaluated by the government and presents the physician's ability and experience.	A dummy variable is used to measure whether a leader is a chief/associate chief physician.
Individual response speed	*IPS*	The response speed of individual service, namely the service of team leader.	Original data
Other services providing	*OServices*	Except for the text consultation service, the leader may also provide other services such as telephone consultation.	A dummy variable is used with 1 presenting that the leader has provided other services.
Working experience online	*Experience*	The experience is measured by the total time that the physician works online.	Its logarithm value
Recommendation	*Rec*	Evaluated by Haodf and reflects physician's medical ability and popularity among patients.	Original data
Departments	*Department*	The departments that the team belongs to.	13 dummy variables are used to measure 14 departments

For the role of price, the empirical models include team-based service price (*TSP*), individual service price (*ISP*), and price difference (*AbsPD*). The individual service refers to the leader's own text consultation service. In general, the team-based service price and the individual service price are different. We calculated the price difference between the individual service price and the team-based service price and used its absolute value to measure the influence of the price gap on the patient decision.

Team characteristics included team-based response speed (*TRS*) and the number of team members (*#Members*) and are used as moderating variables. The response speed (reported in percentages) indicates how many patients can get a reply in 24 h. It is often used as a service quality indicator in existing studies (37, 38), and can influence patients' price perception. The number of team members is used to measure team size.

Control variables were also included in the empirical models, including team information and leader (i.e., individual) information. For information on the team, we referred to the team introduction which was posted on the team's homepage, along with their fields of expertise (*TInt*). For information on the leader, hospital introduction (*HInt*), level of the hospital (*Level*), medical title (*Title*), city location (*City*), individual service response speed (*IRS*), online working experience (*Experience*), recommendation (*Rec*), and provision of other services (*OServices*) were included. These variables generally have an effect on the patient's decision and have been usually considered in existing studies (37, 38). In addition, although we focused on mild diseases from departments such as gynecology and obstetrics, endocrinology, and E.N.T., we also included the department (*Department*) that the team belongs to in the models.

### Statistics analysis

The ordinary least squares (OLS) method was used to obtain the results of the empirical analyses in two steps for the following models:

**First step**


$$\begin{array}{l} \#Demands=\beta_{0}+\beta_{1}TSP+\beta_{2}ISP+\beta_{3}TRS+\beta_{4}\#Members\\ \;+\beta_{5}TSP\times ISP+\beta_{6}TSP\times TRS+\beta_{7}TSP\\ \;\times \#Members+\beta_{8-29}C+\varepsilon \end{array}$$

**Second step**


$$\begin{array}{l} \#Demands=\beta_{0}+\beta_{1}TSP+\beta_{2}AbsPD+\beta_{3}TRS\\ \;+\beta_{4}\#Members+\beta_{5-26}C+\varepsilon \end{array}$$

where ***β*** are the parameters to be estimated, ***ε*** is the error team and ***C*** are the control variables. *TSP*×*ISP, TSP*×*TRS*, and *TSP*×*#Members* are interaction effects. All empirical analyses were done using STATA.

## Results

### Descriptive statistics

Tables 2, 3 show the variable description and correlation matrix. From Table 2, we find that service prices vary considerably among individuals and teams. Moreover, for the lead physician and his team, the price difference also varies widely (max value is 561 CNY) with a mean value of 51.79 CNY. In general, 4–5 physicians formed a team, and 70% of teams could reply in 24 h. On average, the increment of orders of team-based service in 1 month was just 1.56, and there is still ample room for growth. The correlation matrix in Table 3 shows no serious multicollinearity.

**Table 2:** Variable description.

**Variables**	**Min**	**Max**	**Mean**	**Standard deviation**
*#Demands*	0	43	1.56	3.27
*TSP*	9	800	84.47	89.68
*ISP*	6	659	89.44	96.23
*AbsPD*	0.00	561	51.79	60.85
*TRS*	0.00	1.00	0.70	0.352
*#Members*	2	24	4.47	2.81

**Table 3:** Correlation matrix.

**Variables**	**1**	**2**	**3**	**4**	**5**	**6**	**7**	**8**	**9**	**10**	**11**	**12**	**13**	**14**
*Level*														
*Hint*	0.295^**^													
	0.000													
*City*	0.092^**^	0.058												
	0.004	0.068												
*Title*	0.052	0.073^*^	0.064^*^											
	0.103	0.021	0.044											
*TRS*	0.024	−0.053	0.015	−0.073										
	0.556	0.183	0.714	0.068										
*OServices*	0.043	−0.007	−0.058	−0.060	0.156^**^									
	0.178	0.832	0.065	0.058	0.000									
*Experience*	−0.022	−0.022	−0.007	0.014	0.023	0.089^**^								
	0.490	0.488	0.825	0.649	0.568	0.005								
*Rec*	0.216^**^	0.173^**^	0.281^**^	0.021	0.181^**^	0.189^**^	0.014							
	0.000	0.000	0.000	0.510	0.000	0.000	0.664							
*TSP*	0.118^**^	0.157^**^	0.221^**^	0.089^**^	0.124^**^	0.066^*^	−0.041	0.402^**^						
	0.000	0.000	0.000	0.005	0.002	0.038	0.192	0.000						
*IRS*	−0.017	−0.009	0.033	−0.088^**^	0.279^**^	0.214^**^	0.023	0.256^**^	0.181^**^					
	0.608	0.777	0.312	0.008	0.000	0.000	0.476	0.000	0.000					
*Tint*	−0.006	−0.005	0.066^*^	0.042	0.035	−0.012	−0.022	0.076^*^	0.025	0.069^*^				
	0.861	0.863	0.038	0.185	0.385	0.701	0.480	0.017	0.435	0.037				
*#Members*	0.023	−0.021	0.066^*^	0.134^**^	−0.069	0.031	−0.025	0.112^**^	0.078^*^	0.041	0.196^**^			
	0.461	0.503	0.039	0.000	0.085	0.327	0.425	0.000	0.014	0.214	0.000			
*ISP*	0.174^**^	0.179^**^	0.307^**^	0.165^**^	0.079^*^	0.110^**^	−0.031	0.514^**^	0.547^**^	0.099^**^	0.056	0.138^**^		
	0.000	0.000	0.000	0.000	0.050	0.001	0.336	0.000	0.000	0.003	0.077	0.000		
*AbsPD*	−0.084^**^	−0.054	−0.137^**^	−0.102^**^	0.033	−0.062	−0.005	−0.198^**^	0.331^**^	0.066^*^	−0.040	−0.082^**^	−0.609^**^	
	0.008	0.091	0.000	0.001	0.410	0.051	0.881	0.000	0.000	0.046	0.210	0.010	0.000	
*#Demands*	0.113^**^	0.089^**^	0.140^**^	0.035	0.181^**^	−0.009	−0.052	0.349^**^	−0.032	0.063	0.050	0.088^**^	0.263^**^	−0.327^**^
	0.000	0.005	0.000	0.276	0.000	0.788	0.099	0.000	0.319	0.057	0.115	0.006	0.000	0.000

** p < 0.05.

* p < 0.01.

### Empirical results

The analysis in this paper was undertaken in two steps. First, we examined the relationship between team-based service prices, individual service prices, and team-based service demands. And then, the moderating effects of the individual service price and team characteristics were studied. The results are shown in Table 4.

**Table 4:** Empirical results for the first step.

**Variables**	**Model 1**		**Model 2**		**Model 3**	
	**Co-ef. (STD)**	**Sig**.	**Co-ef. (STD)**	**Sig**.	**Co-ef. (STD)**	**Sig**.
*TSP*			−0.282 (0.030)	0.000	−0.206 (0.100)	0.040
*ISP*			0.164 (0.028)	0.000	0.319 (0.082)	0.000
*TRS*			0.291 (0.074)	0.000	−0.395 (0.365)	0.280
*#Members*			0.013 (0.008)	0.075	0.061 (0.036)	0.092
*TSP × ISP*					−0.036 (0.018)	0.043
*TSP × TRS*					0.174 (0.090)	0.050
*TSP × #Members*					−0.011 (0.008)	0.181
Control variables	Yes		Yes		Yes	
Departments	Yes		Yes		Yes	
Adjusted *R*^2^	0.034		0.209		0.214	
*F* change (sig)	13.387 (0.000)		26.015 (0.000)		3.343 (0.019)	

All variables explain the 21.4% variance in *#Demands*, and the values of R Square change are all significant. To test the hypotheses, we conducted three models. Model 1 included just the control variables; independent variables, and moderating variables were included in Model 2 (to examine H1a and H1b), and their interaction terms were introduced in Model 3 (to examine H2, H4a, and H4b).

Model 2 in Table 4 shows that team-based service price is negatively related to patients' team-based service demands (*β* = −0.282, *p* < 0.001), while individual service price positively impacts patients' team-based service demands (*β* = 0.164, *p* < 0.001), thus both H1a and H1b are supported. For the moderating effects of the individual service price and team characteristics, Model 3 shows that individual service price negatively moderates the relationship between team-based service price and demands (*β* = −0.036, *p* < 0.05), and team response speed has a positive moderating effect on the relationship between team-based service price and demands (*β* = 0.174, *p* = 0.05). Thus, H2 and H4a are supported. We did not find a significant moderating effect of team members, so H4b was not supported.

We then investigated the effects of the price difference on service demands. The results are shown in Table 5. By calculating the difference between the individual service price and the team-based service price, we found a negative effect of the price difference on team-based service demands (*β* = −0.085, *p* < 0.05). Accordingly, H3 was supported.

**Table 5:** Results of the price difference.

**Variables**	**Model 1**	
	**Co-ef. (STD)**	**Sig**.
TSP	−0.217 (0.031)	0.000
AbsPD	−0.085 (0.037)	0.021
Control variables	Yes	
Departments	Yes	
Adjusted *R*^2^	0.215	
*F* change (sig)	5.324 (0.021)	

### Robustness check and the *post-hoc* analysis

Based on the definition of a team [a social system of three or more people embedded in an organization or context who collaborate on a common task (11)], we deleted teams with only two physicians and used the new dataset to get empirical results and we got consistent results with our main results (results table is omitted to save space).

While examining the effects of the price difference, we found significant results (see Table 5). To determine if there was a turning point, we checked further and included the square of the price difference in the empirical model. However, there were no significant results (see Table 6). Therefore, our findings suggested that the teams should try to minimize their price gaps with the lead physician service price.

**Table 6:** *Post-hoc* analysis.

**Variables**	**Model 1**	
	**Co-ef. (STD)**	**Sig**.
AbsPD	−0.023 (0.081)	0.774
AbsPD^2^	0.031 (0.037)	0.395
Control variables	Yes	
Departments	Yes	
Adjusted *R*^2^	0.215	
*F* change (sig)	0.725 (0.395)	

## Discussion and implications

### Discussion

Given that some studies have begun to examine the role of price in the online medical service environment (8, 9), limited studies have explored them in both individual and team-based contexts. In an online medical platform, a physician can provide individual service and join a team to provide team-based service, which should raise the question of whether patients' decisions would be influenced by the price of the individual service and the price of the team-based service and how should physicians establish prices for individual service and team-based service? Guided by transaction utility theory, this study investigates the influence of pricing strategy on service demands from the price difference perspective by focusing on two typical service models: individual service and team-based service. Team characteristics (response speed and team size) were also considered. By collecting information on 1,100 teams and 1,100 individual physicians, empirical results support most of our hypotheses. Price fairness provides a proper framework for understanding pricing strategy in individual and team-based services in the online environment. Specifically, for the 14 departments, a negative influence of team-based price has been obtained. As a substitute service, a higher individual service price will make patients turn to team-based service. A big price difference will enhance perceptions of unfair pricing, which is consistent with prior studies (19). Team characteristics also provide a cue for patients and consequently influence their decisions as established in prior studies [e.g., (36)].

#### The individual service price will be regarded as a reference point

Consistent with classical price theory (32), a higher price will decrease the patient's choice. As a team is formed by several physicians, pricing strategy also needs to consider the individual service price. The lead physician is the core member of a team and often attracts more attention from patients, therefore we focused on the influence of the leader. Our results show that individual service price negatively moderates the relationship between team-based service price and demands, which suggests that individual service price will be regarded as a reference point and influences patient price perception. A higher individual service price would increase patients' unfair feelings and decreases their purchase intention.

#### Big price difference undermines patient support

By calculating the price difference between the individual service price and the team-based service price, we found a negative role played by price difference in affecting patient purchase decisions. Based on the medical options on Haodf, lead physicians can provide their individual service and also participate in a team to provide team-based service. Patients can get that information on the physician's homepage, and their price perception would be influenced by reviewing both individual and team service prices. Although prior studies believe price differences will lead to unfair perception (19), our research has proved its effects on purchase decisions by conducting empirical analysis. However, as no turning point was identified, it suggests that teams try to minimize their price gaps with the lead physician's individual service price as best they can.

#### Hard work on improving service quality is always useful

Although price is a crucial factor, there are also other important signals in the market that jointly influences consumer decision. By introducing team characteristics, we found few consistent and inconsistent results with prior studies. For the effect of response speed, quick response attracts more patients as response speed is a key factor that reflects the quality of the service delivery process (38). However, we did not find a significant effect of team size, the reason may be because team size has a dual role. Although more physicians jointly provide medical services and a big team size helps improve diversity, team size has also been proven to be a major antecedent of social loafing or productivity loss in technology-supported teams (40, 41). Therefore, it requires careful selection of team size in actual operation.

### Implications

This study contributes to knowledge in three key ways. First, we used the transaction utility theory and price fairness to frame our conceptual model and contribute to them by introducing a new measurement of the price difference. The price difference is often considered between different sellers (19). In our paper, as a physician can provide different types of service, we could obtain the price difference between the services of a lead physician. In addition, prior studies mainly concentrated on the relationship between price difference and unfair perception and then hypothesized its influence on consumer purchase (19). By collecting a real dataset, we examined the effect of price differences on the consumer's actual purchase behavior. Our results suggest that big differences will undermine patient support and decrease their purchase intention.

Second, this study enriches the literature on online medical platforms from two aspects. Firstly, although researchers have begun to pay attention to the role of price in online medical platforms, what remains to be examined and studied further is quite significant. This study investigated both the role of the price and the role of the price difference. Secondly, studies on online medical platforms often include only one service type in their studies, namely individual service (38) or team-based service (36). This study considered both individual and team-based services and explored their interactions. Our results show that there is an interactive effect between individual and team-based services, and physicians need to consider them to set pricing strategies.

Third, this study extends the understanding of virtual teams. On the one hand, we enrich the literature on virtual teams in healthcare and find that price and team characteristics will influence patient purchase decisions. We also find team characteristics affect patient price perception. On the other hand, our context is unique. In our context, a team member can provide service independently, and also provide service as part of a team. Thus, to a certain extent, individual service and team-based service are substitutable despite being competitive. By introducing the impact of individual-level information, we find team members, especially team leaders, have a great impact on team performance.

This paper also has significant practical contributions. For physicians, our results can help them understand what aspects should be given more attention to improving their service. Based on this research, this study recommends improving competitiveness through setting suitable pricing strategies. The lead physician is the core member of a team and often attracts more attention from patients, therefore physicians need to balance the relationships between leaders and teams. Pricing strategies need to consider both individual service and team-based services. On the one hand, as a higher price will decrease patient choice, it is imperative to keep both individual and team prices within reasonable bounds. On the other hand, given a big price difference decreases patient purchase decisions (though no turning point was found), it is equally important to keep the price difference between individual service and team-based services small. In addition, other indicators also need to be paid attention to, for example, response speed. A quick response attracts more patients as response speed is a key factor that reflects the quality of the service delivery process.

Substantial orders are crucial for the sustainable development of online medical platforms. With the development of the economy, consumers are increasingly seeking a variety of products or services, therefore, the need for a better understanding of the effects of prices from a price difference perspective is important. The influence of price on patient decisions is more complex in service than in product areas. Knowing how price contributes to patient decisions is essential since such knowledge enables the participants in healthcare to configure prices that obtain between levels of performance.

### Limitations and future directions

Although this study makes significant contributions to theory and practice, this paper has several limitations and future directions. First, this paper only includes the role of the lead physician, although the leader is the core of a team, the service prices of other members could also impact patients' decisions. Future studies including information on all team members are needed. Second, we calculated the price difference between individual service and team-based service and included its absolute value in the models. Therefore, our results cannot explain the influences of positive or negative price differences, which can be explored in future research. Third, this study only proposes the negative role of price by collecting non-critical disease data from select departments. It is challenging to judge the severity of diseases by the department. Future studies should use more accurate indicators. Fourth, restricted by the available data, we could not include other important influencing factors of consumer decisions such as patient preference and experience; further research can construct a systematic price-decision model to help understand the role of price in healthcare.

## Data availability statement

The original contributions presented in the study are included in the article/supplementary material, further inquiries can be directed to the corresponding author.

## Author contributions

WL, WW, CL, QL, and LF conceived and designed the study, developed the research model, undertook data collection and analysis, and drafted and modified the manuscript. All authors contributed to this paper. All authors approved the final version of the manuscript for submission.

## Funding

This study was funded by the Hainan Natural Science Foundation of China (No. 821RC578), the Research and Cultivation Fund of Hainan Medical University (No. HYPY2020025), and Hainan Province Clinical Medical Center (No. QWYH202175).

## Conflict of interest

The authors declare that the research was conducted in the absence of any commercial or financial relationships that could be construed as a potential conflict of interest.

## Publisher's note

All claims expressed in this article are solely those of the authors and do not necessarily represent those of their affiliated organizations, or those of the publisher, the editors and the reviewers. Any product that may be evaluated in this article, or claim that may be made by its manufacturer, is not guaranteed or endorsed by the publisher.
